# Multimodal imaging in a case of bilateral astrocytic hamartoma with retinitis pigmentosa

**DOI:** 10.3205/oc000209

**Published:** 2023-01-30

**Authors:** Sugandha Goel, Debmalya Das, Kumar Saurabh, Rupak Roy

**Affiliations:** 1 Department of Vitreo Retina, Aditya Birla Sankara Nethralaya, Kolkata, India

**Keywords:** astrocytic hamartoma, multicolor imaging, retinitis pigmentosa

## Abstract

Astrocytic hamartoma is a benign glial tumor. It may be associated with tuberous sclerosis and can also be found incidentally on retinal examination as an isolated presentation. Here, we describe multimodal imaging characteristics of astrocytic hamartoma in a patient with retinitis pigmentosa. Spectral domain optical coherence tomography of both eyes showed moth-eaten optically empty spaces and hyperreflective dots along with foveal thinning. Multicolor image highlighted mulberry appearance of the lesion with green shift signifying elevated lesion. In infrared reflectance, lesion was hyporeflective with its margins well delineated. Green reflectance and blue reflectance highlighted calcification as multiple hyperreflective dots. Autofluorescence showed typical hyperautofluorescence.

## Introduction

Astrocytic hamartoma is a benign and stable tumor that originates from glial astrocytes of the retina or optic nerve. It may be associated with phakomatosis like tuberous sclerosis and neurofibromatosis or found incidentally on retinal examination as an isolated presentation [1]. A rare association has been found in patients with retinitis pigmentosa (RP) [2], [3], [4]. Here, we describe multimodal imaging characteristics of astrocytic hamartoma in a patient with retinitis pigmentosa.

## Case description

**Figure 1:**
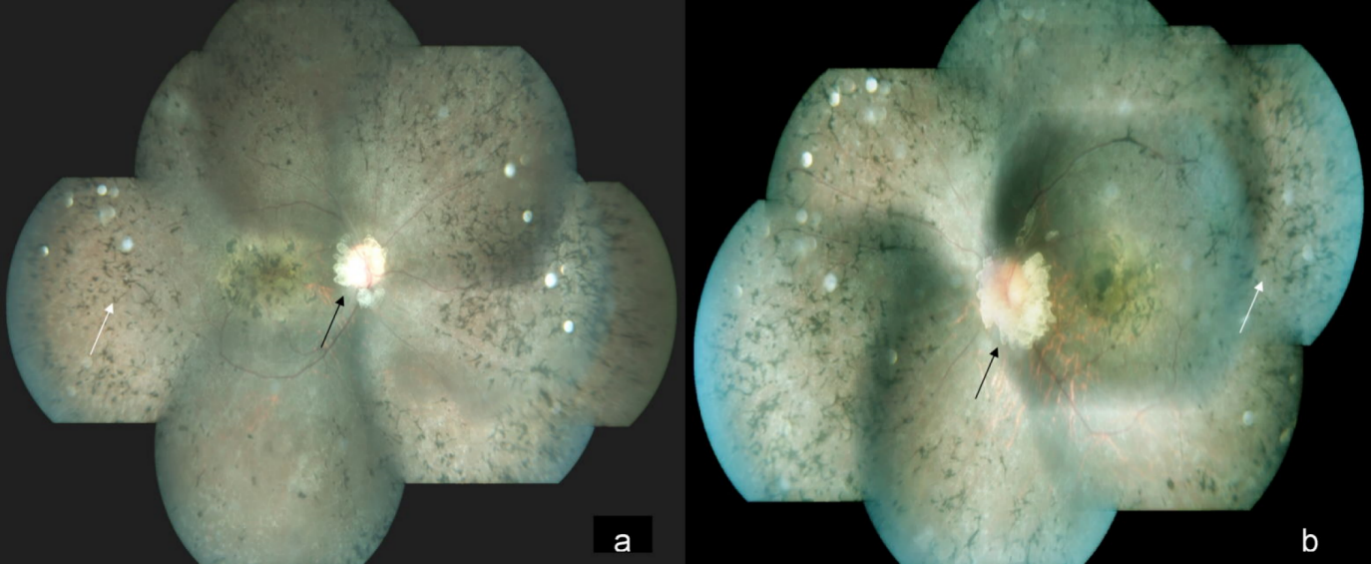
Color fundus photograph of the right eye (a) and the left eye (b) showing a creamy white, semitranslucent, elevated lesion surrounding the optic disc (a and b) (black arrow) along with bony spicules (a and b) (white arrow) in the mid periphery

**Figure 2:**
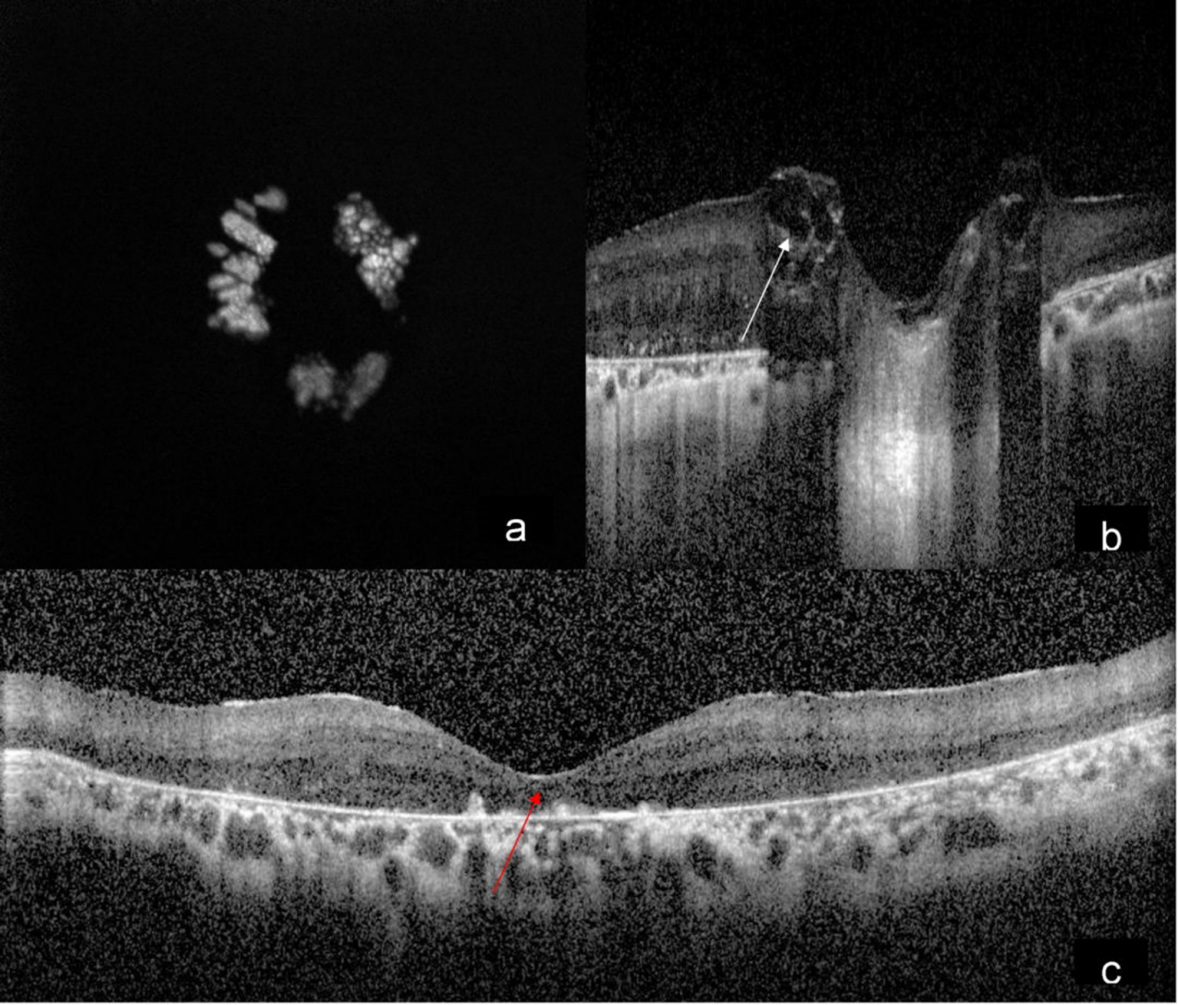
Autofluorescence of the right eye (a) showing typical hyperautofluorescence; SD-OCT showing moth eaten optically empty spaces (b) (white arrow) and hyperreflective dots along with foveal thinning (c) (red arrow)

**Figure 3:**
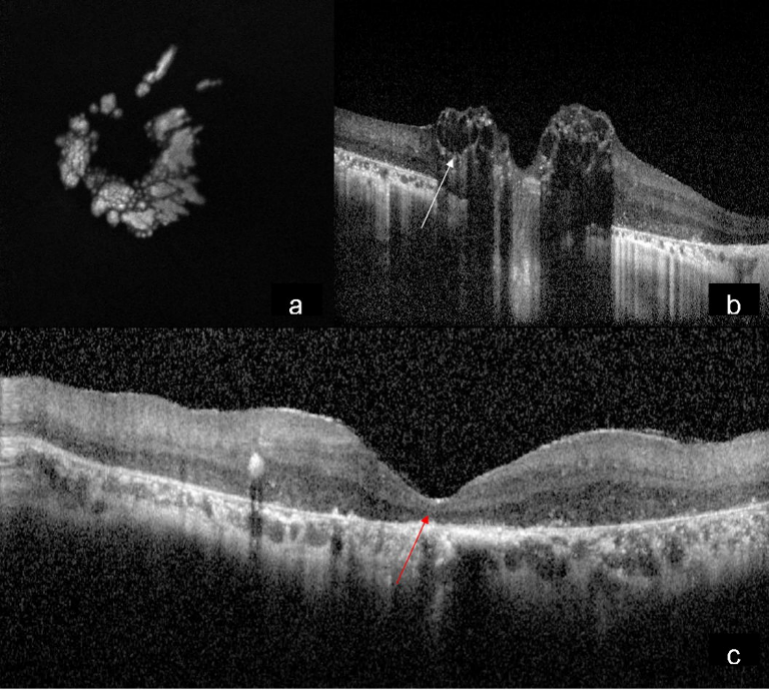
Autofluorescence of the left eye (a) showing typical hyperautofluorescence; SD-OCT showing moth eaten optically empty spaces (b) (white arrow) and hyperreflective dots along with foveal thinning (c) (red arrow)

**Figure 4:**
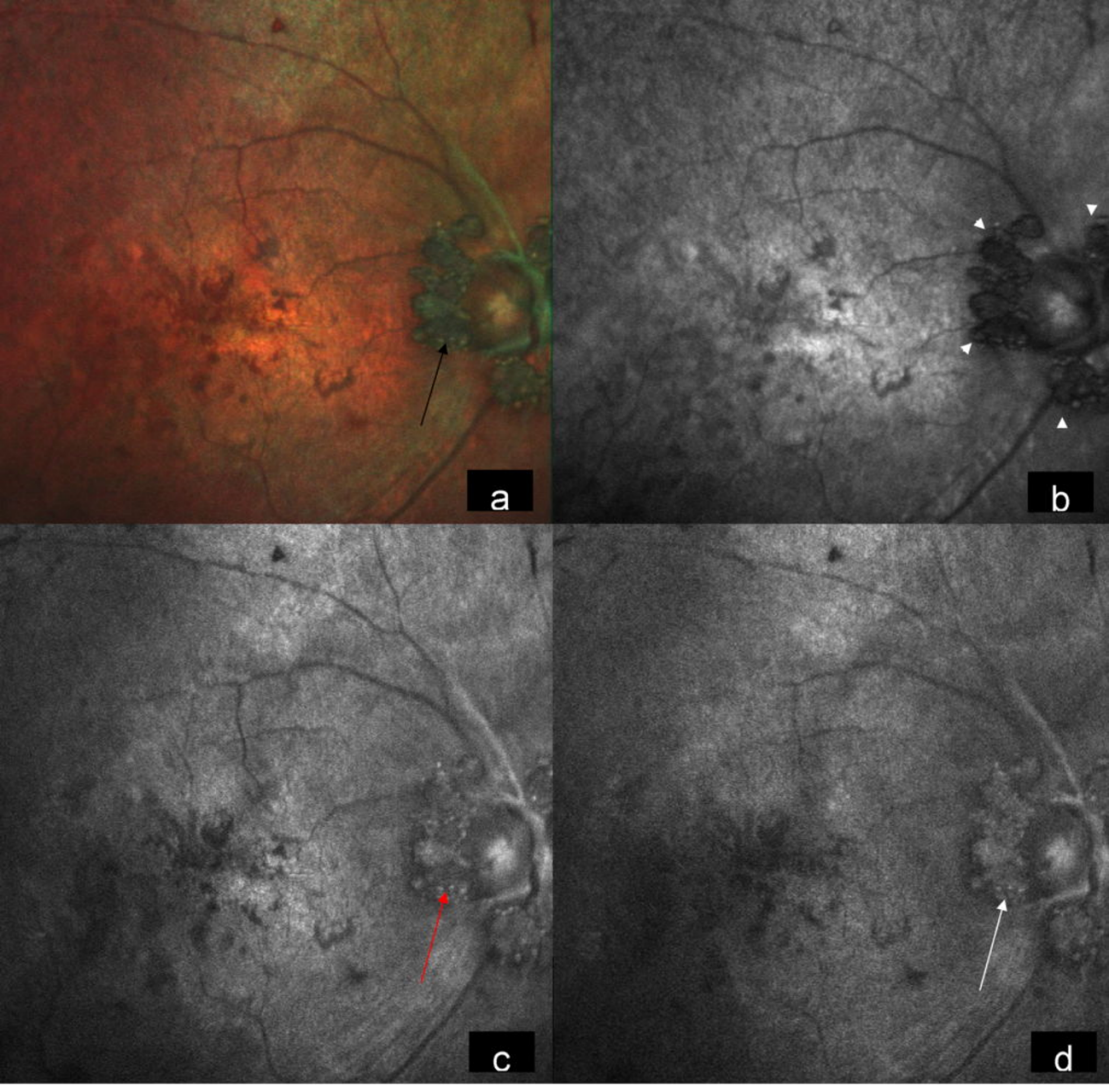
Multicolor image of the right eye highlighting mulberry appearance of the lesion with green shift (a) (black arrow). In Infrared reflectance, the lesion is hyporeflective with its margins well visualized (b) (arrow heads). Green reflectance (c) (red arrow) and blue reflectance (d) (white arrow) show multiple hyperreflective dots.

**Figure 5:**
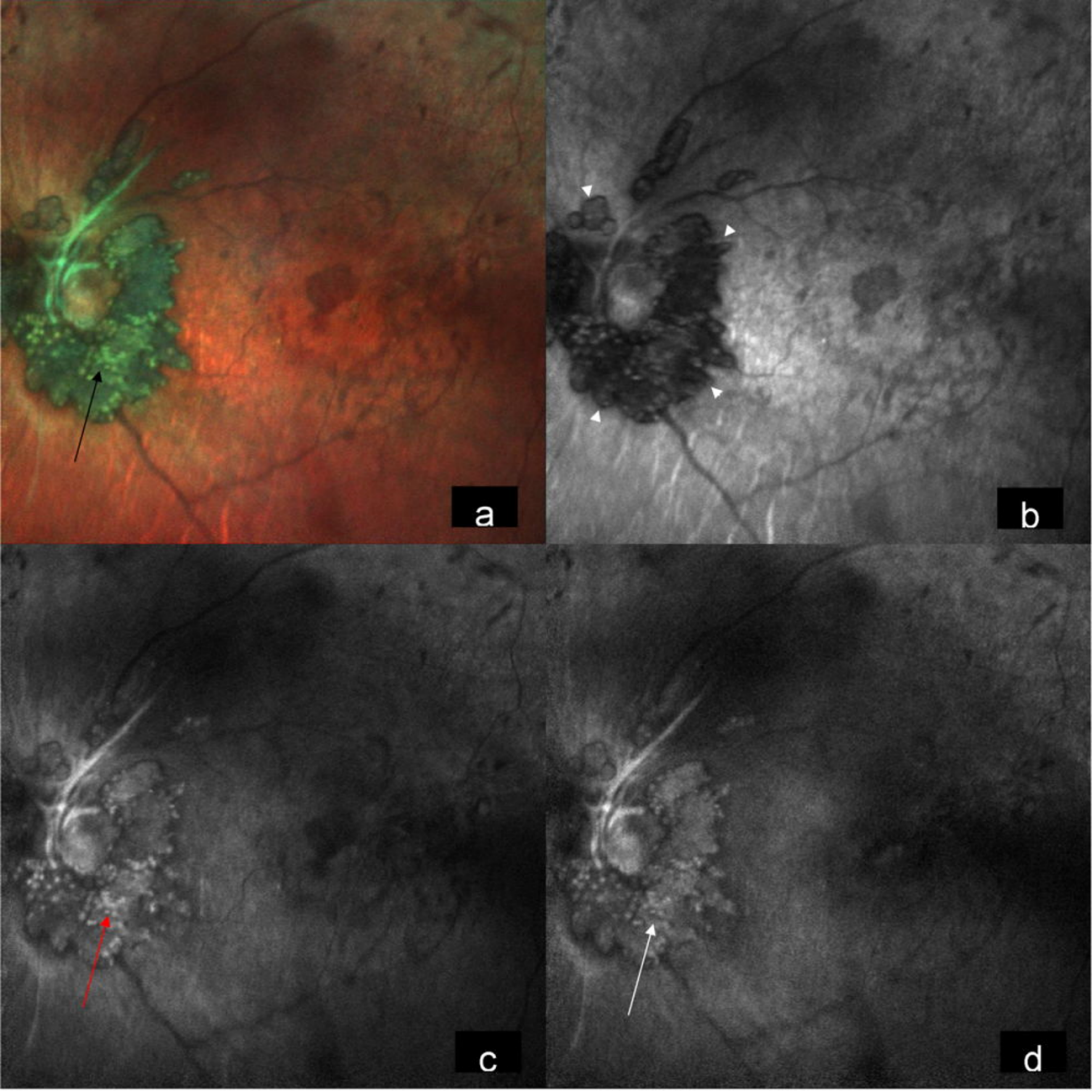
Multicolor image of the left eye highlighting mulberry appearance of the lesion with green shift signifying elevated lesion (a) (black arrow). In infrared reflectance, the lesion is hyporeflective with its margins well visualized (b) (arrow heads). Green reflectance (c) (red arrow) and blue reflectance (d) (white arrow) show multiple hyperreflective dots.

A 15-year-old male came to our hospital with gradual diminution of vision and night blindness in both eyes since 5 years. There was no family history of phakomatosis. Comprehensive examination did not reveal clinical features of phakomatosis. Best-corrected visual acuity was 20/1200 and 20/600 in right and left eye, respectively. Fundus examination of both eyes showed creamy white, semitranslucent, elevated lesion surrounding the optic disc along with attenuation of blood vessels and bony spicules in the mid-periphery (Figures 1a/b (Fig. 1)). Spectral domain optical coherence tomography showed moth-eaten optically empty spaces and hyperreflective dots (Figure 2b (Fig. 2) and Figure 3b) along with foveal thinning (Figure 2c (Fig. 2) and Figure 3c (Fig. 3)). Autofluorescence imaging of the optic nerve head (Figure 2a (Fig. 2) and Figure 3a (Fig. 3)) showed hyperautofluorescence. Multicolor image highlighted mulberry appearance of the lesion with green shift (Figure 4a (Fig. 4) and Figure 5a (Fig. 5)). In infrared reflectance (IR), margins of the lesion were better visualized (Figure 4b (Fig. 4) and Figure 5b (Fig. 5)). Green reflec-tance (GR) (Figure 4c (Fig. 4) and Figure 5c (Fig. 5)) and blue reflectance (BR) (Figure 4d (Fig. 4) and Figure 5d (Fig. 5)) showed multiple hyperreflective dots suggestive of calcification. A diagnosis of astrocytic hamartoma with RP was made.

## Discussion

Astrocytic hamartoma should be monitored closely to look for any progression. It should be differentiated from other optic nerve head lesions like optic nerve head meningioma, hemangioma, papilledema, combined hamartoma of the retina and retinal pigment epithelium, and most commonly optic nerve head drusen [3]. Multicolor imaging in retinal astrocytoma has been recently described in the literature [5]. Multicolor imaging provides topographic evidence of tissue, a feature which is not permissible with conventional white light colour photography. Our case showed a green shift in multicolor composite image signifying the elevated nature of the lesion. Intralesional calicification was dramatically highlighted as multiple tiny glistening yellow dots characteristic of astrocytic hamartoma. BR and GR were particularly effective in picking up intralesional calcification. One of the primary aims of imaging in these cases is to pick up tumor growth at an early stage. Proper delineation of tumor margin assumes great importance in this regard. In our case, tumor margins were better visualized in both multicolor image and IR image.

## Conclusion

This report highlights characteristics of multicolor imaging in astrocytic hamartoma and its association with RP.

## Notes

### Competing interests

The authors declare that they have no competing interests.
